# mRNA-seq reveals skeletal muscle atrophy in response to handling stress in a marine teleost, the red cusk-eel (*Genypterus chilensis*)

**DOI:** 10.1186/s12864-015-2232-7

**Published:** 2015-12-01

**Authors:** Jorge E. Aedo, Jonathan Maldonado, Víctor Aballai, Juan M. Estrada, Macarena Bastias-Molina, Claudio Meneses, Cristian Gallardo-Escarate, Herman Silva, Alfredo Molina, Juan A. Valdés

**Affiliations:** Laboratorio de Biotecnología Molecular, Facultad de Ciencias Biológicas, Universidad Andrés Bello, Santiago, Chile; Laboratory of Biotechnology and Aquatic Genomics, Universidad de Concepción, Concepción, Chile; Interdisciplinary Center for Aquaculture Research (INCAR), P.O. Box 160-C, Concepción, Chile; Centro de Investigación Marina Quintay (CIMARQ), Universidad Andrés Bello, Quintay, Chile; Departamento de Producción Agrícola, Laboratorio de Genómica Funcional & Bioinformática, Universidad de Chile, Facultad de Ciencias Agronómicas, Av. Santa Rosa 11315, La Pintana, 8820808 Santiago, Chile; Centro de Biotecnología Vegetal, Facultad Ciencias Biológicas, Universidad Andrés Bello, Santiago, Chile

**Keywords:** *Genypterus chilensis*, Red cusk-eel, mRNA-seq, Handling stress, Skeletal muscle atrophy, Cortisol

## Abstract

**Background:**

Fish reared under intensive conditions are repeatedly exposed to stress, which negatively impacts growth. Although most fish follow a conserved pattern of stress response, with increased concentrations of cortisol, each species presents specificities in the cell response and stress tolerance. Therefore, culturing new species requires a detailed knowledge of these specific responses. The red cusk-eel (*Genypterus chilensis*) is a new economically important marine species for the Chilean aquaculture industry. However, there is no information on the stress- and cortisol-induced mechanisms that decrease skeletal muscle growth in this teleost.

**Results:**

Using Illumina RNA-seq technology, skeletal muscle sequence reads for *G. chilensis* were generated under control and handling stress conditions. Reads were mapped onto a reference transcriptome, resulting in the *in silico* identification of 785 up-regulated and 167 down-regulated transcripts. Gene ontology enrichment analysis revealed a significant up-regulation of catabolic genes associated with skeletal muscle atrophy. These results were validated by RT-qPCR analysis for ten candidates genes involved in ubiquitin-mediated proteolysis, autophagy and skeletal muscle growth. Additionally, using a primary culture of fish skeletal muscle cells, the effect of cortisol was evaluated in relation to red cusk-eel skeletal muscle atrophy.

**Conclusions:**

The present data demonstrated that handling stress promotes skeletal muscle atrophy in the marine teleost *G. chilensis* through the expression of components of the ubiquitin-proteasome and autophagy-lysosome systems. Furthermore, cortisol was a powerful inductor of skeletal muscle atrophy in fish myotubes. This study is an important step towards understanding the atrophy system in non-model teleost species and provides novel insights on the cellular and molecular mechanisms that control skeletal muscle growth in early vertebrates.

**Electronic supplementary material:**

The online version of this article (doi:10.1186/s12864-015-2232-7) contains supplementary material, which is available to authorized users.

## Background

Over the past decades, the aquaculture industry has significantly advanced in terms of technology and rearing methods due to the growing demand of fish for human consumption [1]. While many different species are cultured worldwide, the Chilean finfish industry is highly concentrated in salmonid farming [2, 3]. However, there is a recent local and global trend towards diversifying breeding species to maintain the sustainability of the aquaculture industry [4, 5]. In Chile, one such cultivated marine species is the red cusk-eel (*Genypterus chilensis*, Guichenot, 1881), a teleost of the *Ophidiidae* family [6]. This fish is highly valued in national and international markets due to exceptional flesh quality and high nutritional value [7, 8]. Nevertheless, the culturing of this species is seriously hindered by scarce biological knowledge and, primarily, limited information on the negative effects of stress associated with intensive farming in marine species [9].

The stress response in fish occurs when a stimulus is perceived as a threat. This response increases cortisol secretion, as mediated through the hypothalamic-pituitary-interrenal axis [10]. If the stress is chronic, affected individuals could have permanently increased circulating cortisol levels, a situation that involves short-term metabolic changes and a long-term response associated with reduced growth [11]. An important tissue for growth regulation is skeletal muscle, which is fundamental for an organism’s metabolism and physiology [12, 13]. In mammals, it is well documented that under stressful pathological conditions increased cortisol levels are associated to skeletal muscle atrophy results from increased protein breakdown and decreased protein synthesis [14], however relatively little is known about this condition in teleost skeletal muscle. The two major route that increases overall rates of protein degradation during muscle atrophy are the ubiquitin-proteasome and the autophagy-lysosome systems [15]. The stimulation by cortisol of these two protein degradation pathways are mediated through the increased expression of several atrogenes, such as Atrogin-1 [16], Foxo [17] and MURF-1 [18], as well as other intracellular mediators related to autophagy such as KLF15 [19], and REDD1 [20]. Although a few studies have gone further in the understanding of skeletal muscle response in relation to different stressors in teleosts by using PCR arrays [21, 22], microarray [23–26], and RNA-seq approaches [27–30], there are no studies focused to understand the relevance of cortisol in the skeletal muscle response to stress.

It was only recently that the first annotated transcriptome of *G. chilensis* was published through the use of Illumina technology, thus providing valuable transcriptomic information for this species and for members of the *Ophidiidae* family [31]. The aim of the present study was to characterize the effect of the stress caused by handling on the skeletal muscle of *G. chilensis,* an economically important marine fish in the Chilean aquaculture industry. For this, Illumina reads obtained from control and stressed fish were mapped onto the reference transcriptome of the red cusk-eel to identify differentially expressed transcripts (DETs). Gene ontology enrichment analysis revealed a significant up-regulation of catabolic genes associated with skeletal muscle atrophy. Using *in vitro* approximations, it was also determined that cortisol-mediated genomic actions are a powerful inductor of skeletal muscle atrophy in fish. In the short term, these results will aid in modifying rearing protocols, the aim of which is to improve animal welfare. Importantly, these results also contribute towards the overall understanding of the atrophy system in a non-model teleost, providing novel insights on the cellular and molecular mechanisms that control skeletal muscle growth in early vertebrates.

## Results

### Physiological and transcriptomic responses of red cusk-eel skeletal muscle to handling stress

**Fig. 1:**
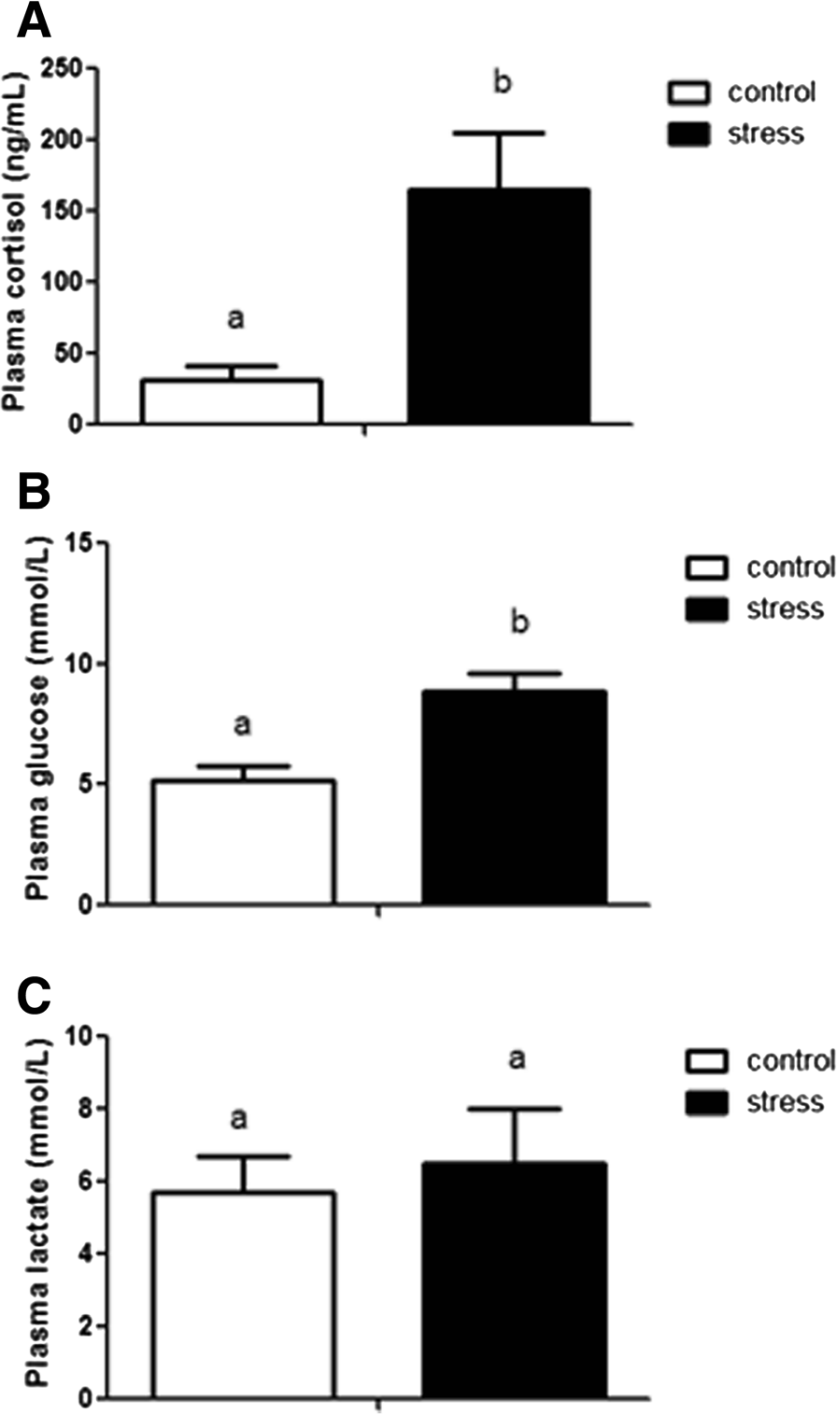
Plasma cortisol, glucose and lactate levels in control and handling stress groups. Data are represented as means ± SEM (*n* = 4). For all graphs, white and black bars represent control and stressed groups respectively. Different letters indicate significant differences among sampling points of each group

Juvenile red cusk-eels were stressed daily with a netting and chasing protocol, and samples were obtained after five days of this procedure. Plasma cortisol and glucose levels significantly increased (5.3-fold and 1.7-fold, respectively) as compared to the control fish, revealing the magnitude of the stress induced by handling. However, no significant differences in plasma lactate levels were observed (Fig. 1). Two separate cDNA libraries were constructed from the skeletal muscle of pooled juvenile red cusk-eels from control and stressed conditions. To examine sequencing variations, cDNA library replicates of each condition were constructed. In total, Illumina MiSeq sequencing generated 23,731,898 paired-end reads (Table 1). Raw data were deposited in the NCBI Sequence Read Archive under Accession Number [GenBank SRS614525: SRR2060847, SRR2063782, SRR2064146, SRR2064148]. After trimming adapters, low quality base pairs, and short reads, the two sequence sets were reduced to 22,992,184 high-quality reads (Table 1). These reads were analyzed with the CLC Genomic Workbench software v.7.0.3 using the previously reported reference transcriptome for *G. chilensis* [31], resulting in ~98.8 % of the reads mapped. The expression level of each transcript was represented as RPKM, with 785 transcripts up-regulated under stressed conditions and 167 transcripts down-regulated under stressed conditions. A complete list of the differentially expressed transcripts is included in Additional file 1: Table S1.

**Table 1:** Summary of sequencing and mapping results

Condition	Number of reads	Average length	Number of reads after trimming	Average length after trimming	Percent of mapped reads	Percent of mapped reads with functional anotation
Control	5,369,110	157.2	5,261,728	153.1	98.9	43.4
Control (replicate)	6,475,266	152.4	6,268,257	151.9	99.1	43.5
Handling stress	5,037,796	170.2	4,488,662	157.5	98.7	43.3
Handling stress (replicate)	6,849,726	165.5	6,575,737	156.2	98.5	43.2
Total/average	23,731,898	161.3	22,992,184	154.7	98.8	43.4

### Gene ontology (GO) enrichment, KEGG pathway analysis and qPCR validation

The DAVID gene functional classification tool was used to identify groups of transcripts sharing common GO terms. The transcripts up-regulated under stressed conditions were significantly enriched in biological processes, such as modification-dependent macromolecule catabolic process (GO:0043632), modification-dependent protein catabolic process (GO:0019941), and cellular protein catabolic process (GO:0044257), among others (Table 2). The down-regulated transcripts under stressed conditions were significantly enriched in biological processes including striated muscle contraction (GO:0006941), muscle organ development (GO:0007517), and muscle contraction (GO:0006936), among others (Table 2). The GO terms for up-regulated transcripts were significantly enriched in molecular functions, such as ubiquitin-protein ligase activity (GO:0004842), acid-amino acid ligase activity (GO:0016881), and small conjugating protein ligase activity (GO:0019787), among others (Additional file 2: Table S2). The GO terms for down-regulated transcripts were significantly enriched in molecular functions, such as cytoskeletal protein binding (GO:0008092), structural constituent of muscle (GO:0008307), actin binding (GO:0003779), among others (Additional file 2: Table S2). The enrichment of up-regulated transcripts in cellular component distribution were in membrane-enclosed lumen (GO:0031974), organelle lumen (GO:0043233), and intracellular organelle lumen (GO:0070013), among others (Additional file 3: Table S3). The enrichment of down-regulated transcripts in cellular component distribution were in contractile fiber part (GO:0044449), sarcomere (GO:0030017), and contractile fiber (GO:0043292), among others (Additional file 3: Table S3). Finally, pathway analysis through KEGG revealed up-regulated transcripts to be enriched in ubiquitin-mediated proteolysis, regulation of autophagy, and Proteasome (Additional file 4: Table S4). The down-regulated transcripts were enriched in KEGG pathways such as muscle contraction, hypertrophic cardiomyopathy, and dilated cardiomyopathy (Additional file 4: Table S4).

**Table 2:** Enriched biological processes of up-regulated and down-regulated transcripts in response to handling stress

GO ID	GO term	*p*-value	Fold enrichment
	Enrichment of up regulated transcripts in skeletal muscle under stress		
GO:0043632	Modification-dependent macromolecule catabolic process	2.3E-06	3.8
GO:0019941	Modification-dependent protein catabolic process	2.3E-06	3.8
GO:0044257	Cellular protein catabolic process	3.6E-06	2.7
GO:0008104	Protein localization	6.8E-06	2.8
GO:0015031	Protein transport	7.4E-06	2.8
GO:0045184	Establishment of protein localization	7.4E-06	3.4
GO:0051603	Proteolysis involved in cellular protein catabolic process	9.1E-06	3.4
GO:0044265	Cellular macromolecule catabolic process	1.2E-05	3.2
GO:0046907	Intracellullar transport	1.4E-05	3.3
GO:0030163	Protein catabolic process	3.0E-05	3.1
GO:0009057	Macromolecule catabolic process	8.4E-05	2.8
GO:0006396	RNA processing	3.3E-04	2.8
GO:0006511	Ubiquitin-dependent protein catabolic process	4.4E-03	3.0
	Enrichment of down regulated transcripts in skeletal muscle under stress		
GO:0006941	Striated muscle contraction	1.9E-06	4.3
GO:0007517	Muscle organ development	4.3E-06	4.2
GO:0006936	Muscle contraction	5.5E-06	5,7
GO:0003012	Muscle system process	2.3E-05	3.5
GO:0014706	Striated muscle tissue development	3.1E-05	4.9
GO:0060537	Muscle tissue development	5.8E-05	6.7
GO:0048738	Cardiac muscle tissue development	6.6E-05	6.7
GO:0043462	Regulation of ATPase activity	9.5E-05	3.5
GO:0060048	Cardiac muscle contraction	2.5E-03	4.2
GO:0006937	Regulation of muscle contraction	6.5E-03	3.1

**Fig. 2:**
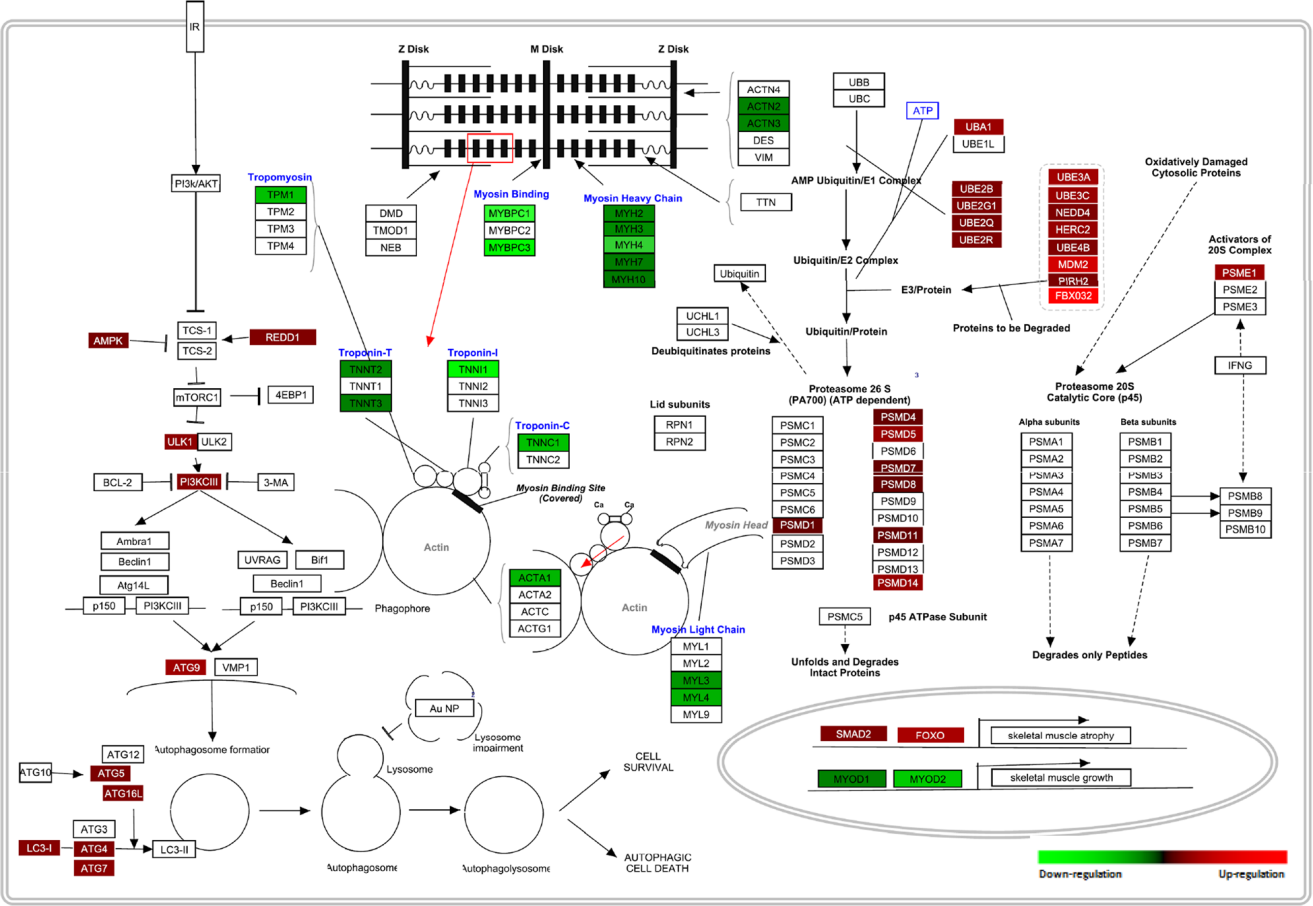
Comprehensive illustration of transcription changes in skeletal muscle of red cusk-eel under handling stress using modified KEGG pathway maps using Pathvisio v3. The figure depicts ubiquitin-proteasome. autophagy-lysosome and striated muscle contraction genes (indicated as rectangles)

PathVisio 3.0 was used to visualize and integrate the transcriptomic data obtained in KEGG pathway analysis, related to ubiquitin-mediated proteolysis, regulation of autophagy, and muscle contraction (Fig. 2). 21 transcripts associated to the ubiquitin-mediated proteolysis were up-regulated such as components of the E1- ubiquitin activating enzyme (*uba1*), E2-ubiquitin conjugating enzyme (*ube2b*, *ube2g1*, *ube2q*, *ube2r*), E3-ubiquitin ligase (*ube3a*, *ube3c*, *nedd4*, *herc2*, *ube4b*, *mdm2*, *pirh2*, *fbx032*), as well as components of the 26S proteasome (*psmd1*, *psmd4*, *psmd5*, *psmd7*, *psmd8*, *psmd11*, *psmd14*, *psme1*). Similarly, 10 transcripts associated to the regulation of autopaghy were up-regulated such as components of mTOR signaling (*ampk*, *redd1*, *ulk1*, *pi3kciii*) and components that control autophagosome formation (*atg9*, *atg5*, *atg16l*, *atg4*, *atg7*, *lc3i*). Oppositely, 17 transcripts associated to muscle contraction were down-regulated such as components of the Z-disk (*actn2*, *actn3*), myosin heavy chain (*myh2*, *myh3*, *myh4*, *myh7*, *myh10*), myosin binding (*mybpc1*, *mybpc3*), tropomyosin (*tpm1*), troponin (*tnnt2*, *tnnt3*, *tnni1*, *tnnc1*), actin chain (*acta1*), and myosin light chain (*myl3*, *myl4*). Additionally, transcription factors involved in skeletal muscle growth (*myod1*, *myod2*) and transcription factors involved in skeletal muscle atrophy (*smad2*, *foxo1*) were incorporated.

**Fig. 3:**
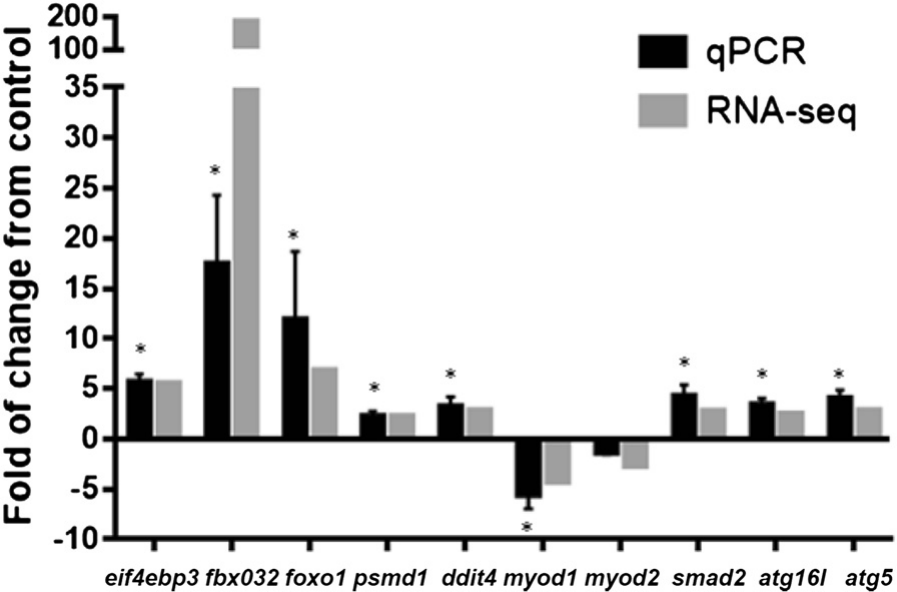
Quantitative real time PCR validation of ten DETs. The transcript expression fold changes measured by RNA-seq and qPCR are indicated by dark grey and light grey columns, respectively. Asterisks on the qPCR values indicate significant differences between control and stressed fish at *p* < 0.05 (*n* = 4)

Eight up-regulated transcripts related to skeletal muscle atrophy (*foxo1*, *ddit4*, *psmd1*, *smad2*, *fbx032*, *eif4ebp3*, *atg5*, and *atg16l1*) and two down-regulated transcripts related to skeletal muscle growth (*myod1* and *myod2*) were selected for RT-qPCR analysis so as to validate the results of RNA-seq analysis. The transcript expression fold-changes measured by these two methods (Fig. 3) were highly correlated, with a significant R^2^ value of 0.85 (*p*-value = 1E^−4^). Taken together, these results indicate that stress induces red cusk-eel skeletal muscle atrophy and suggest that cortisol modulates the expression of components from the ubiquitin-proteasome and autophagy systems.

### Cellular and molecular response of red cusk-eel myotubes to cortisol

**Fig. 4:**
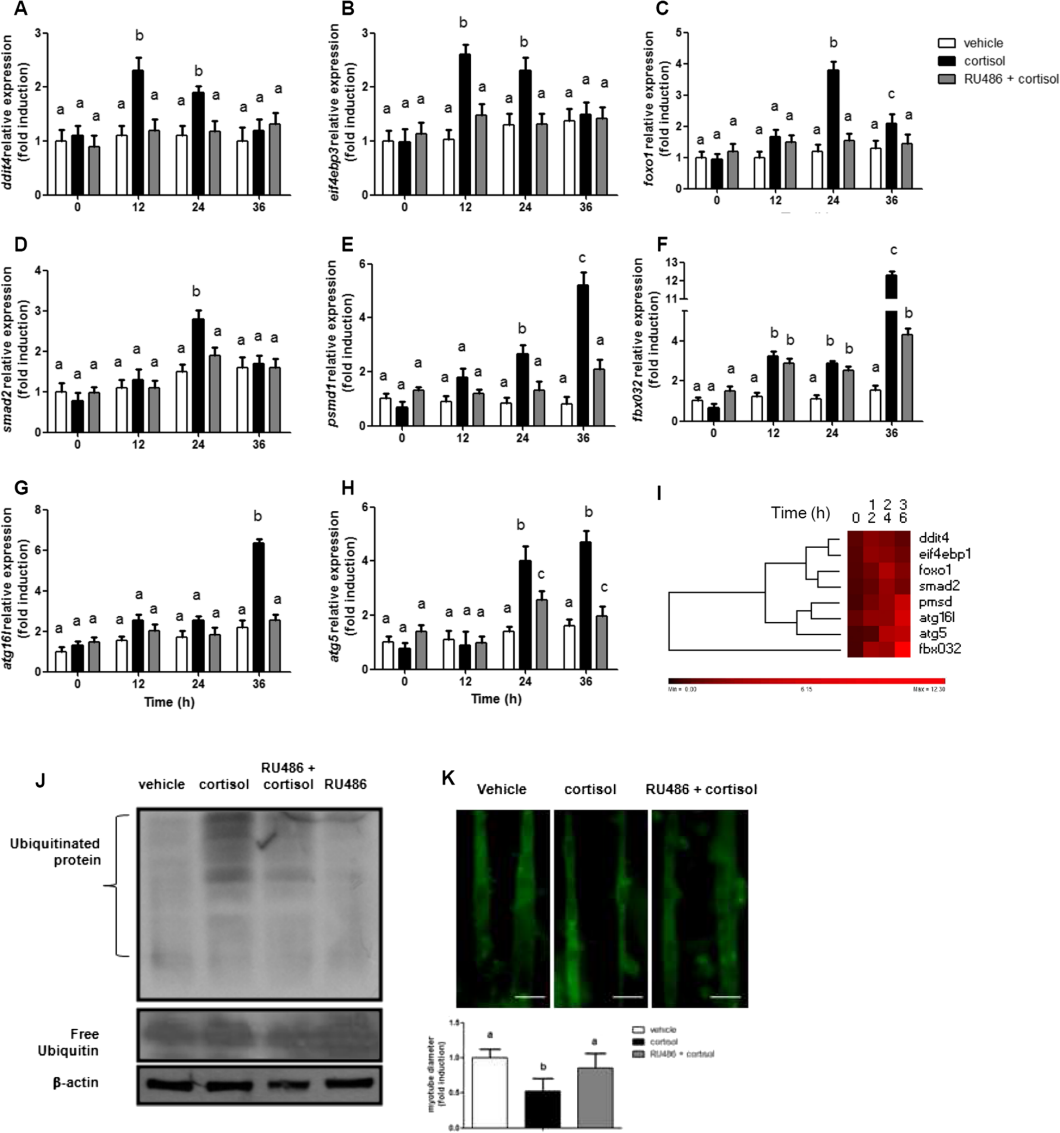
Cortisol induces red cusk eel myotubes atrophy by genomic mechanism. **a**–**h** *eif4ebp3, fbx032, foxo1, psmd1, ddit4, smad2, atg16l* and *atg5* expression in red cusk-eel myotubes stimulated with cortisol or RU486-cortisol for each indicated times. mRNA levels were analyzed by RT-qPCR and showed as a relative expression normalized with respect to *fau*. Data are represented as means ± SEM of duplicates from 3 independent experiments and are expressed as fold change relative to values in control cells. **i** Heat map summary and hierarchical clustering of the components of the atrophy system in the skeletal muscle of the red cusk-eel. In the heat map, the red color indicates an increase of the components of the atrophy system. **j** Western blot showing protein ubiquitination and total ubiquitin in red cusk-eel myotubes treated with vehicle, cortisol, RU486-cortisol or RU486. Protein extract was obtained 36 h after stimulation. **k** Myotubes diameter measurement expressed as a percentage of the diameter in the control group. Red cusk-eel myotubes were incubated with vehicle, cortisol or RU486-cortisol. Analyses were performed 5 days after stimulation. Different letters indicate significant differences among sampling points of each group. Scale bar equals 50 μm

To determine if cortisol was the main modulator of skeletal muscle atrophy in red cusk-eel, the mRNA expression of *foxo1*, *ddit4*, *psmd1*, *smad2*, *fbx032*, *eif4ebp3*, *atg5*, *atg16l, myod1* and *myod2* were monitored in myotube lysates 12, 24, and 36 h following cortisol treatment (250 ng/mL) (Fig. 4a-i). At 12 h post-treatment, maximum increases in *ddit4* and *eif4ebp3* mRNA expressions were observed. At 24 h post-treatment, there was a maximum increase in *foxo1* and *smad2* mRNA expressions, which was followed by maximum increases of *psmd1*, *fbx032*, *atg5*, and *atg16l* mRNA expressions at 36 h post-treatment with cortisol. Pretreatment of skeletal myotubes with the glucocorticoid receptor antagonist RU486 (1 μM) significantly inhibited the cortisol-induced up-regulation of *foxo1*, *ddit4*, *psmd1*, *smad2*, *fbx032*, *eif4ebp3*, *atg5* and *atg16l*. No changes in gene expression were observed for *myod1* or *myod2* following cortisol treatment (data not shown). To determine whether cortisol treatment effectively induced atrophy in fish myotubes, protein ubiquitination levels were recorded 36 h post-treatment. Increased ubiquitination occurred after treatment, and this increase was inhibited by pretreatment with RU486 (Fig. 4j). Additionally, cortisol treatment triggered a significant decrease in myotube diameters, in association with muscular atrophy, at five days post-treatment (Fig. 4k). These results indicate that cortisol, through their genomic action, is a powerful inductor of skeletal muscle atrophy in fish myotubes.

## Discussion

*Genypterus chilensis* is an economically important marine species for Chilean aquaculture industry. The first reference transcriptome for this species was previously sequenced and annotated through Illumina HiSeq paired-end sequencing [31]. In this study, RNA-seq analysis was used to determine the effects of handling stress on the skeletal muscle of the red cusk-eel, revealing 952 DETs associated with an up-regulation of catabolic biological processes or a down-regulation of skeletal muscle contraction biological processes. The results obtained by this *in silico* approach were validated by a RT-qPCR analysis of genes representative of the most relevant identified processes, as well as by an *in vitro* approach that revealed interesting details regarding the regulation dynamics and mechanisms of cortisol-mediated skeletal muscle atrophy in fish. In general terms, the present study found the primary stress response associated to increased levels cortisol and glucose in plasma was very similar to other fish species, such as the rainbow trout (*Oncorhynchus mykiss*) [32] or Senegalese sole (*Solea senegalensis*) [33].

In teleosts, cortisol is the primary glucocorticoid and regulator of the physiological response to stress [34]. Cortisol binds and activates specific corticoid receptors in target tissues. In fish there are two types of corticoid steroid receptors: mineralocorticoid receptors (MR) and glucocorticoid receptors (GR) [35]. Most fish possess one MR and two GR isoforms, GR1 and GR2 [36]. Once inside the cell, cortisol binds to cytoplasmic GRs to induce conformational changes that cause the receptor to dissociate from respective chaperone molecules. The hormone-receptor complex then translocates to the nucleus where it dimerises and binds to the glucocorticoid response elements of target genes [37]. Depending on the co-factors recruited, this leads to the transactivation or transrepression of these genes. This mode of cortisol action is referred to as the classical or genomic pathway [37]. Cortisol-mediated stress is a key controller in aerobic and anaerobic metabolism [38], increasing gluconeogenesis, and inhibiting glycogen synthesis [39]. An increased metabolic rate contributes to reduced growth. Cortisol may also control growth by acting through elevated plasma glucose levels to reduce appetite and food ingestion [40]. Due to this, elevated cortisol levels could be inferred through reduced food conversion efficiency, and growth [40].

For years it was assumed that the low growth rates experienced by fish under stressful conditions were due to metabolic changes impacting anabolic processes [41]. However, the present work is the first to evidence that stress mediated by cortisol induces the expression of components associated with skeletal muscle catabolism. The transcripts up-regulated under stressed conditions were associated with catabolic pathways such as ubiquitin-mediated proteolysis and autophagy, while down-regulated transcripts were associated with muscle contraction or muscle hypertrophy pathways. Interestingly in humans, similar signatures have been observed in skeletal muscle atrophy induced by corticosteroids used to treat several pathological conditions or by cortisol released in response to stress conditions such as sepsis, cachexia, and starvation [42].

In mammals, *in vitro* models have been very useful in defining the glucocorticoid pathway by reducing complexity, as compared to an *in vivo* model [43, 44]. Similarly, fish myotubes primary culture has been used as a valid model for understanding the signaling pathways involved in muscle development and growth in teleost, including for glucose transport [45], TOR signaling [46], IGF-1 signaling [47–49], Myostatin signaling [50, 51], GH signaling [52], proinflammatory cytokine signaling [53], PAMPs signaling [54], and cortisol signaling [55]. Similarly, in the present study using an *in vitro* model, it was determined that cortisol through the expression of components of the ubiquitin-proteasome and autophagy-lysosome systems was an inductor of skeletal muscle atrophy in fish,.

The maximum *foxo1*expression in myotubes occurred 24 h post-treatment with cortisol. The role of this transcription factor in skeletal muscle atrophy induced by glucocorticoids has been demonstrated in mammals through *in vitro* and *in vivo* approximations [56–58]. Moreover, a recent study demonstrated the presence of functional glucocorticoid response elements in the mammalian FoxO1 promoter [59]. In fish, the expression and activity of FoxO1 have been observed under nutritional stress in fine flounder (*Paralichthys adspersus*) [60]. Interestingly, IGF-1, an inductor of skeletal muscle hypertrophy, induced the phosphorylation of FoxO1 and FoxO4 in trout myotubes in association with decreased transcriptional activity [61]. The described FoxO1 target genes included atrogin-1 (*fbx032)*, a protein involved in muscle proteolysis via the ubiquitin-proteasome system [17]. Atrogin-1 expression occurs in relation to skeletal muscle atrophy under fasting conditions in rainbow trout [62] and fine flounder [60]. Recently, it was determined that FOXO1/Atrogin-1 signaling pathway is involved in the skeletal muscle atrophy induced by LPS in rainbow trout myotubes [54].

Other important components of protein catabolism include the 26S proteasome and autophagosome. The up-regulation of the 26S proteasome non-ATPase regulatory subunit 1 (*psmd1*) has been observed in atrophying fast-twitch muscles from rats treated with dexamethasone [63]; however, there are no reports on the differential expression of this gene in teleosts. A similar phenomenon has been reported for ATG5 and ATG16L1, where dexamethasone treatment in L6 myotubes induces the expression of several autophagy genes 24 h post-treatment, including of *atg5* and *atg16l1* [64]. In teleost species, ATG5 activity in zebrafish has been linked to neurogenesis and organogenesis [65]. Nevertheless, there are no reports that relate ATG5 gene expression with fish muscle atrophy.

Another gene with differential expression identified in this study was REDD1 (*ddit4*), a cytoplasmic protein with a crucial role in repressing protein synthesis mediated by TOR [66]. Additionally, the same signaling pathway showed an over expression of 4EBP-1 (*eif4ebp3*). This protein represses protein synthesis by directly interacting with the eukaryotic translation initiation factor 4E [66]. In both cases, the present study detected maximum expression 12 h post-treatment with cortisol, suggesting that the inhibition of protein synthesis precedes atrogene expression. In mammals, acute dexamethasone treatment induces REDD1 mRNA expression in rat skeletal muscle *in vivo* and in L6 myoblasts, as well as down-regulating mTOR signaling through the activation of 4EBP-1 [67]. In zebrafish, REDD1 regulates dorsoventral patterning through the negative modulation of Wnt/β-catenin activity [68].

Among the genes down-regulated under handling stress, the present study found proteins associated with contractile functions and development, including specific muscle transcription factors such as MyoD1 and MyoD2. Both proteins belong to the myogenic regulatory factors (MRFs) protein family and are involved in the regulation of teleost myogenesis [69]. While both transcripts had decreased expression in RNA-seq analysis, only *myod1* showed a significant decrease in its expression in RT-qPCR analysis. Moreover, changes in expression were not detected for *myod1* or *myod2* in fish myotubes treated with cortisol. This result differs from that observed in mammals, where treatment of the C2C12 myoblast with dexamethasone reduces *myod* expression [70]. This observation can be explained by the differences between mammalian and teleost myogenesis [71].

## Conclusions

The present work used RNA-seq analysis to determine the effects of handling stress on the skeletal muscle transcriptome of *G. chilensis*, an important marine fish for the Chilean aquaculture industry. Handling stress induced physiological changes, associated with increases in circulating levels of cortisol, and major changes in global skeletal muscle gene expression. Under stressed conditions, 785 transcripts were up-regulated in association with catabolic signaling pathway such as the ubiquitin-proteasome and autophagy-lysosome systems. Conversely, 167 transcripts were down-regulated under stressed conditions in association with biological processes such as muscle contraction and muscle system processes. The transcriptional expression of components from the ubiquitin-proteasome and autophagy-lysosome pathways were analyzed by RT-qPCR and all were significantly increased under handling stress. To further analyze the relevance of cortisol in teleost skeletal muscle atrophy, an *in vitro* approximation was performed, providing details about the dynamics and mechanisms by which cortisol induces skeletal muscle atrophy in teleosts. There was a coordinated expression of genes related to the suppression of protein synthesis, protein ubiquitination, and autophagy. All of these were modulated by the genomic actions of cortisol. This is the first study to indicate that stress and cortisol are powerful inductors of skeletal muscle atrophy in a teleost, in addition to providing valuable information for monitoring the culturing and growth of marine fish species under intensive rearing conditions.

## Methods

### Fish sampling and stress conditions

Juvenile red cusk-eels (*Genypterus chilensis*) with an average weight of 900 ± 50 g and length of 55 ± 5 cm were collected from the Centro de Investigación Marina de Quintay (CIMARQ) (33°13′S 71°38′W, V Region, Valparaíso, Chile). Fish were maintained under natural temperature and light:dark photoperiod conditions (13 °C ± 1 °C and L:D 12:12) for the spring season. The specimens were randomly distributed between a control and stressed group, placed in two separate 90 L tanks, and acclimated for 2 weeks before the experiment. The stressed group was then subjected to a standardized handling stress protocol consisting of netting and chasing the fish for 5 min daily for 5 days [32]. Six hours after the final handling stimulation, stressed and control fish were quickly netted, and blood samples were taken via caudal puncture using heparinized tubes (*n* = 4 per group). Plasma was collected by centrifugation at 5000 × *g* for 10 min and stored at −80 °C until analysis. Following blood sampling, fish were sacrificed through an overdose of anesthetic (3-aminobenzoic acid ethyl ester, 300 mg/L), and white muscle was collected, immediately frozen in liquid nitrogen, and stored at −80 °C until analysis. Fish were reared and sampled according to protocols approved by the Bioethical Committee of Andrés Bello University.

### Cortisol, glucose, and lactate measurements

Plasma cortisol levels were measured using the Enzyme Immunoassay Kit (Cayman, MI, USA) following the manufacturer’s recommendations. Glucose and lactate plasma levels were measured using Colorimetric Assay Kits (Abcam, Cambridge, UK) following the manufacturer’s recommendations.

### Skeletal muscle RNA extraction and transcriptome sequencing

Total RNA was extracted using the RNeasy Mini Kit (Qiagen, TX, USA) following the manufacturer’s instructions. RNA was quantified through spectrophotometry using NanoDrop technology with the Epoch Multi-Volume Spectrophotometer System (BioTek, VT, USA). Total RNA isolated from skeletal muscle was treated with DNase I to remove genomic DNA. RNA concentration was mesuared by Qubit® 2.0 Fluorometer (Life Technology, Carlsbad, CA, USA) and RNA integrity was determined using Fragment Analyzer™ Automated CE System (Analytical Advanced Technologies, Ames, IA, USA). Library construction of 1 μg total RNA for each sample was made using Illumina® TruSeq® RNA Sample prep kit (Illumina®, USA), according to protocol indications, with mRNA fragmentation time of 2 min at 94 °C. After PCR amplification step, mRNA libraries sizes were verified on AATI Fragment Analyzer™, and quantified by qPCR-based quantification using Kapa Library Quantification kit (#KK4824). Libraries were diluted and prepared to a final concentration of 12,5pM for loading on Illumina® MiSeq desktop sequencer, according to the MiSeq System User guide. cDNA library replicates of each condition were constructed and sequenced.

### Data processing, differentially expressed transcripts, and GO enrichment analysis

Raw sequencing reads were trimmed by removing Illumina adapter sequences and low quality bases. Sequences shorter than 50 base pairs were also discarded. To analyze *in silico* gene expression levels, the CLC Genomics Workbench v.7.0.3 software (http://www.clcbio.com/genomics/) was used with the *G. chilensis* reference transcriptome [31]. RNA-seq analysis was carried out for sequence reads obtained from control and stressed conditions using the following default parameters: two mismatches, minimum fraction length of 0.9, minimum fraction similarity of 0.8, and a maximum of 10 hits per read. Gene expressions were based on reads per kilobase of exon model per million mapped reads (RPKM) values [72]. The up- and down-regulated genes were analyzed for gene ontology (GO) and Kyoto Encyclopedia of Genes and Genomes (KEGG) pathways through the DAVID database [73] and categorized based on GO terms for biological processes, molecular functions, and cellular components, as well as by genes up- or down-regulated in association with KEGG pathways. To establish a relationship between red cusk-eel DETs and DAVID background, a tBLASTx search was conducted against *Danio rerio* Ensembl proteins for significant matches with the red cusk-eel transcriptome. *Danio rerio* Ensembl Gene IDs were obtained from the corresponding Ensembl protein entries. DAVID analysis was performed with custom IDs set as the ‘Background’. Standard settings for gene count (2) and ease (0.1) were used. The cut-off *p*-value for biological processes was 1E^−6^ and cut-off *p*-value for molecular functions and cellular components was 1E^−3^.

### Cell cultures and cortisol treatments

Primary myoblasts were isolated from juvenile red cusk-eels. Dorsal white muscle was obtained under sterile conditions and placed in an F10 medium containing 9 mM NaHCO_3_, 20 mM HEPES, 15 % horse serum, 100 U/ml penicillin, and 10 mg/ml streptomycin at pH 7.4. After mechanical dissociation of the muscle, the tissue was digested with a 0.1 % collagenase and 0.1 % trypsin solution in an F10 medium for 4 h at 15 °C. The suspension was centrifuged at 300 × *g* for 5 min at 10 °C, and the resulting pellet was resuspended in an F10 medium. The cellular suspension was filtered through 40 μm nylon filters. The cells were centrifuged at 1000 × *g* for 10 min at 15 °C, resuspended in 3 ml of cold PBS, and layered on top of 4 ml of Ficoll-Paque gradient (GE Healthcare) in a 15 ml tube. Samples were then centrifuged at 1400 × *g* for 30 min at 15 °C. Following this, the cell layer was extracted with a pipette and washed with 10 ml of F10 medium. Cells were seeded at a density of 2 × 10^6^ per mL in plates previously treated with poly-L-lysine and laminin. Cells were incubated for seven days at 15 °C under atmospheric air in a proliferating medium containing the F10 medium, 9 mM NaHCO_3_, 20 mM HEPES, 10 % fetal bovine serum, 100 U/ml penicillin, and 10 mg/ml streptomycin. Then, myoblasts were cultivated an additional seven days in a differentiating medium composed by the F10 medium, 9 mM NaHCO_3_, 20 mM HEPES, 100 U/ml penicillin, and 10 mg/ml streptomycin to obtain differentiated myotubes. On the fourteenth day, myotubes were treated with a medium containing either ethanol (vehicle control), cortisol (250 ng/mL), or RU486 (1 μM). After 12, 24, and 36 h, the medium was removed, and cells were harvested for total RNA isolation or protein extraction. All treatments were performed with *n* = 4, in two independent experiments.

### Real-time qPCR

Total RNA was extracted from skeletal muscle tissue and skeletal myotubes using the RNeasy Mini Kit (Qiagen, TX, USA) following the manufacturer’s recommendations. RNA was quantified by spectrophotometry using NanoDrop technology with the Epoch Multi-Volume Spectrophotometer System (BioTek, VT, USA). Only RNAs with an A260/280 ratio between 1.9 and 2.1 were used for cDNA synthesis. Residual genomic DNA was removed using the genomic DNA wipeout buffer included in the Quantitect Reverse Transcription Kit (Qiagen, TX, USA). Subsequently, 1 μg of RNA was reverse transcribed into cDNA for 30 min at 42 °C using the manufacturer’s recommendations. qPCR analysis was performed with the Stratagene MX3000P qPCR System (Stratagene, CA, USA). Each qPCR reaction mixture contained 7.5 μl of 2× Brilliant II SYBR Master Mix (Stratagene, CA, USA), 6 μl of cDNA (diluted 40-fold), and 250 nM of each primer. For gene expression normalization, the reference gene 40S ribosomal protein S30 (*fau*) was used [74]. The primers used in this study are listed in Additional file 5: Table S5. Control reactions included a no template control and a control without reverse transcriptase. The QGene program was used for analysis of gene expression [75].

### Western blot analysis

After treatment, cells were solubilized at 4 °C in 30 μL of lysis buffer containing 50 mM Tris-HCl pH 7.4, 150 mM NaCl, 1 mM EDTA, 1 % NP-40, 5 mM Na_3_VO_4_, 20 mM NaF, 10 mM sodium pyrophosphate, and a protease inhibitor cocktail (Calbiochem). Proteins extracts were resolved by 10 % SDS-PAGE, transferred to polyvinylidene difluoride membranes (Millipore, MA, USA), and blocked for 1 h at room temperature in Tris-buffered saline, 0.1 % Tween 20, and 5 % fat-free milk. Incubations with primary antibodies (1:1000) were performed at 4 °C overnight. After incubation for 1 h with horseradish peroxidase (HRP)-conjugated secondary antibodies (1:2000), membranes were developed through enhanced chemiluminescence (Amersham Biosciences, Amersham, UK). The films were scanned, and the ImageJ program was employed for densitometric analysis of the bands [76]. Antibodies against ubiquitin (cat. no. 2880), β-actin (cat. no. 4967), and HRP-conjugated secondary anti-rabbit and anti-mouse were obtained from Cell Signaling Technology (MA, USA).

### Myotube diameter measurements

Myotubes were loaded with 5.4 mM Calcein AM for 45 min at 15 °C in an F10 medium. Myotubes were transferred to the recording chamber and mounted on an inverted fluorescence microscope. Fluorescence was detected using excitation at 488 nm and emission at 540 nm, and images were collected. The diameters were measured for a total of 50 myotubes from random fields using the ImageJ program [76]. Myotubes were measured at three points along their length, and results were expressed as a percentage of the diameter in the control group (*n* = 8) in two independent experiments.

### Pathway visualization and Heat map summary of atrophy data

The construction of pathway maps of ubiquitin-proteasome, autophagy-lysosome and striated muscle contraction and visualization of differentially expressed gene were performed using PathVisio v3 [77]. The appropriate gene database was selected, and the expression dataset was created after importing the saved input file using the Import Expression Dataset tab.

The relationships among relative expression profiles, a heat map summary and hierarchical clustering analysis of gene expression was performed using Permutmatrix [78]. Clustering and seriation were based on Pearson’s correlation coefficient of z-score normalized relative transcript abundance values. McQuitty’s method as hierarchical clustering was used.

### Statistical analysis

Data are expressed as the mean ± SE. Differences in means between groups were determined using one-way ANOVA followed by Bonferroni’s post-test. Data were accepted as significant at a value of *P* < 0.05. Correlations between RNA-seq and RT-qPCR data were assessed through multiple linear regression, which obtained the coefficient of determination (R^2^) and *p*-value. All statistical analyses were performed using the program GraphPad Prism v.5.00 (GraphPad Software, CA, USA).

## Additional files

Additional file 1: Table S1. Complete list of up-regulated and down-regulated transcripts in response to handling stress. (XLSX 66 kb) Additional file 2: Table S2. Enriched Molecular functions of up-regulated and down-regulated transcripts in response to handling stress. (XLSX 10 kb) Additional file 3: Table S3. Enriched cellular component of up-regulated and down-regulated transcripts in response to handling stress. (XLSX 10 kb) Additional file 4: Table S4. Enriched KEGG pathways of up-regulated and down-regulated transcripts in response to handling stress. (XLSX 10 kb) Additional file 5: Table S5. Primer sequences for qPCR assay, amplicon size and PCR. (XLSX 10 kb)

## Abbreviations

FPKM: Fragments per kilobase of exon model per million mapped reads
GO: Gene ontology
DET: Differentially expressed transcript
